#  Long-term Morphine-treated Rats are more Sensitive to Antinociceptive Effect of Diclofenac than the Morphine-naive rats 

**Published:** 2013

**Authors:** Esmaeil Akbari, Ebrahim Mirzaei, Naghi Shahabi Majd

**Affiliations:** a *Department of Physiology and Pharmacology, School of Medicine, Mazandaran University of Medical Sciences, Sari, Iran.*; b *School of Pharmacy, Mazandaran University of Medical Sciences, Sari, Iran. *

**Keywords:** Diclofenac sodium, Morphine-dependent rat, Formalin test

## Abstract

This study investigates the effectiveness of the antinociceptive effects of diclofenac, an NSAID, on the nociceptive behavior of morphine-treated rats on formalin test.

Rats were treated with morphine-containing drinking water for twenty one days, which induced morphine dependence. The antinociceptive effects of 8, 16, and 32 mg/kg doses of diclofenac were then evaluated and compared with distilled water in a formalin-based model of pain.

Diclofenac potentiated pain suppression in morphine-dependent rats during the interphase of the formalin test and reduced the pain score during phase II. The post-test analysis revealed that both 16 mg/kg (p < 0.0001) and 32 mg/kg (p < 0.0001) doses of diclofenac had a significant effect on the interphase, while 8 mg/kg (p < 0.05), 16 mg/kg (p < 0.05), and 32 mg/kg (p < 0.01) doses of diclofenac significantly affected phase II. In contrast, the antinociceptive effects of diclofenac on morphine-naïve rats were observed during phase II only with the a 32 mg/kg dose (p < 0.05).

In general, these results suggest that the long-term use of morphine in rats increases their sensitivity to the antinociceptive effects of diclofenac. Furthermore, the results support the existence of a non-opioid-dependent mechanism of pain suppression during the interphase of formalin test.

## Introduction

Morphine and other opioids are among the most commonly known analgesic drugs. These drugs effectively alleviate pain both in standardized animal pain models and in various clinical conditions. Empirical studies have shown that when animals are exposed to opioids over a long period of time, their sensitivity to pain is increased as measured by nociception models such as hyperalgesia or allodynia (1, 2). Clinical evidence in humans has also suggested that the chronic or high-dose consumption of narcotics either by patients who are suffering from chronic pain or by opiate abusers results in hyperalgesia in addition to eliciting opiate dependence (3- 5). Additionally, it has been reported that post-operative pain intensity is higher and longer-lasting in patients who are physically dependent on opiates (6). Furthermore, clinical and experimental evidence has shown that chronic exposure to opioids leads to development of tolerance to opioid-induced analgesia (7, 8).

Thus, pain management in patients with a long history of opiate use is considered to be a medical complication because the strategy of using a higher dose of opiates in such individuals is not only ineffective in alleviating their pain but could also increase their tolerance, hyperalgesia, and dependence (7, 9-11). Opioid-related side effects such as respiratory depression are also more likely to occur in these patients (12). Therefore, the use of non-opioid analgesics such as non-steroidal anti-inflammatory drugs (NSAIDs) (13) and antidepressants, which have multiple analgesic mechanisms, is a logical treatment for such cases.

Diclofenac sodium is an NSAID that non-selectively inhibits the cyclo-oxygenase (COX) pathway and reduces the production of prostaglandins in peripheral tissues (14), which prevents the lowering of the excitation threshold of nociceptors during tissue damage. Previous studies have also identified additional mechanisms that are involved in analgesic action of diclofenac in both peripheral and central nervous systems (15-19). In addition to its antinociceptive effect in animal pain models (20, 21), the systemic administration of diclofenac is widely used in various clinical pain states (22-24). 

Although the long-term exposure to opiates can lead to development of tolerance to opioid-induced analgesia, previous studies have not shown that pain control could occur by administration of the non-opioid analgesics in opiate abusers or opioid-dependent animals. In this study, we investigated the dose-dependent antinociceptive effects of diclofenac sodium in morphine-dependent rats during the formalin test.

## Exprimental

*Animals*

Male Wistar rats (250-300 g) were obtained from the Pasteur Institute of Iran. The animals were housed in groups of four rats per cage under constant conditions of light (12 h light-dark cycles, lights on at 07:00) and temperature (22 ±2°C) with free access to food and water. All experiments were carried out according to the ethical guidelines for the investigation of experimental pain (25). The study protocol was approved by the Animal Care and Use Committee of Mazandaran University of Medical Sciences.

*Drugs*

The drugs used in this study were diclofenac sodium (Sigma, USA), naloxone (Sigma, USA), morphine sulphate (Temad, Iran), formalin (37% formaldehyde), and sucrose (Merck, Germany). To prepare the desired doses, for injection morphine, naloxone and formalin were dissolved in a 0.9% saline solution, and diclofenac was dissolved in distilled water. Oral morphine and sucrose were dissolved at various concentrations in tap water. 

*The formalin test for nociception*

For the formalin test, 50 μl of 2.5% formalin solution was injected to the dorsal surface of the left hind paw. The animal was then moved to a plexiglass container (30x30x45 cm). Through a 45°-angled mirror that was mounted underneath the container, the animal’s nociceptive behavior was observed and recorded over a period of 60 minutes. As previous studies have suggested (26-28), behaviors related to nociception were recorded every 15 seconds according to the following scale: 0= the animal places the injected paw flat on the ground (*i.e*., it feels no pain); 1= the animal places the injected paw partially on the ground (*i.e*., it does not place all its weight on the paw); 2= the animal keeps the injected paw completely off the ground; and 3= the animal licks, bites, or shakes the injected paw. Ultimately, the mean pain scores for phase I (0-6 min), the interphase (9-18 min), and phase II (21-60 min) were separately calculated for each animal and used for statistical analysis. 

*Induction of morphine dependence*

Morphine dependence in rats can be induced by several methods (29-32). This study utilized the oral of morphine intake (33), which was accomplished by adding it to the animals› drinking water. This mimics the development of opioid dependence in humans because the animal can adjust the level of morphine intake itself (29). An oral method was used to induce morphine dependence (33) in which rats take in progressive doses of morphine in their drinking water over a period of twenty-one days. The concentration of morphine that was included in the drinking water was 0.1 mg/cc on the first and second days, 0.2 mg/cc on the third and fourth days, 0.3 mg/cc on the fifth and sixth days, and 0.4 mg/cc from days seven through twenty-one. To minimize the bitter taste of the solution, 5% sucrose was added to the drinking water. To verify that the rats had become dependent, naloxone (2 mg/kg) was injected on day twenty, and the frequency of behaviors that are related to withdrawal symptoms such as jumping, grooming, scratching, and penis-licking was recorded for thirty minutes.

*Experimental protocol*

One week after the rats had adapted to the laboratory environment, they began to drink water that contained morphine and sucrose for twenty-one days and were weighed every other day. On the twentieth day, signs related to withdrawal were observed for thirty minutes to confirm the morphine dependence. The animals were then returned to their home cages and were once again given free access to food and water that contained morphine and/or sucrose. On day twenty-one, the animals were randomly assigned to one of four groups (one control group and three drug-treated groups, n = 4 or 6). Then, thirty minutes before to the start of the formalin test, each group of rats received either a dose of diclofenac sodium (8, 16, or 32 mg/kg) or distilled water that was injected IP in a total volume of 0.5 cc. The doses of diclofenac that were used in this study have not previously been shown to have any effect on locomotion (34). The same procedure was performed on the rats that had been treated for twenty one day with water containing only 5% sucrose (morphine-naïve rats). All experiments were carried out between 14:00 and 17:00. Immediately after undergoing the formalin test, the animals were sacrificed by an overdose of ether.

*Data analysis*

The nociception score of the control group and the diclofenac-treated groups in morphine-treated or -naïve rats were analyzed the three phases of formalin test by one-way analysis of variance (ANOVA). If any significant statistical difference was revealed, a Tukey’s post-test was carried out for multiple comparisons. Data related to withdrawal symptoms in treated and untreated rats were analyzed using a Student›s t-test. The relevant data were presented as the mean ± SEM, and the significance was limit set at p < 0.05. 

## Results

Neither weight reduction nor mortality was observed in either the morphine-treated or the naïve rats during the treatment period (data not shown).

Following a twenty-day intake of drinking water containing either morphine+sucrose or sucrose alone, an withdraw-related intrapertoneal (IP) injection of naloxone was given to the rats, and behaviors related to withdrawal were assessed. A Student›s t-test was used to compare the rats that were treated with morphine+sucrose and those treated with sucrose alone. The results indicated that the withdrawal signsthe morphine-treated animals were significantly higher than in control animals (data not shown). Thus, the oral model used in this study successfully induced morphine dependence. 

*The antinociceptive effects of diclofenac in morphine-dependent or -naïve rats during phase I of the formalin test*

**Figure 1:**
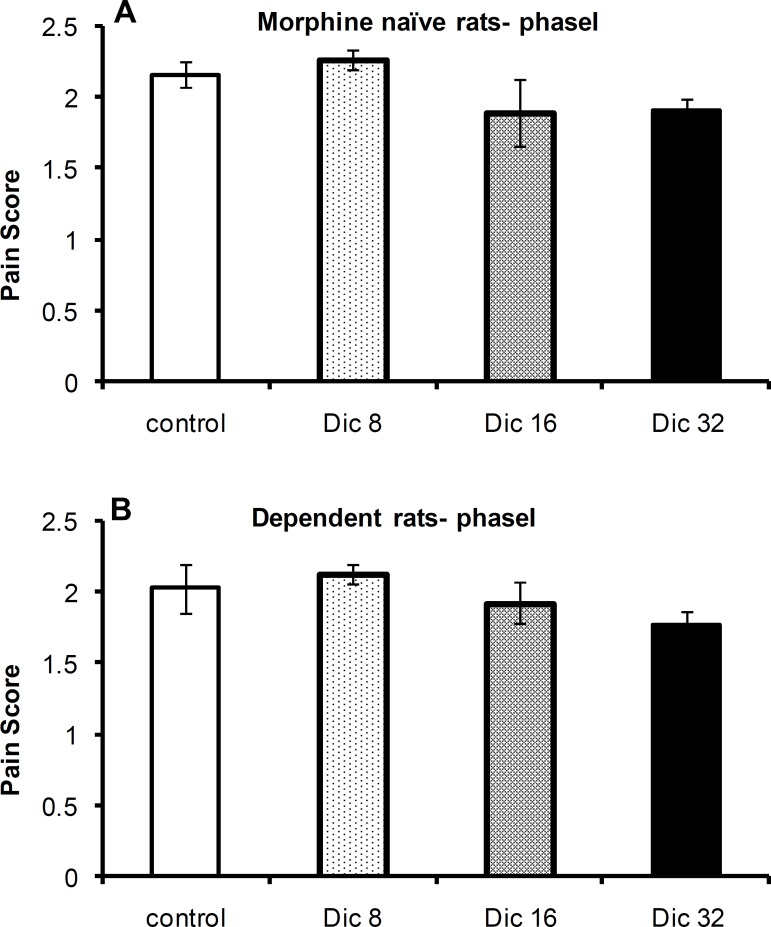
Pain scores during phase I of the formalin test in morphine-naïve (A) and morphine-dependent (B) groups. The data are expressed as the mean±SEM

A one-way ANOVA revealed no significant difference in pain scores among the control group and diclofenac-treated groups in morphine-treated [F(3, 23)=1.43; p = 0.26; n = 6] or -naïve [F(3,21)=1.46; p = 0.26; n = 6 or 4] rats during phase I of the formalin test (Figures 1A and 1B, respectively). 

*The antinociceptive effects of diclofenac in morphine-dependent or -naïve rats during the interphase of the formalin test*

**Figure 2:**
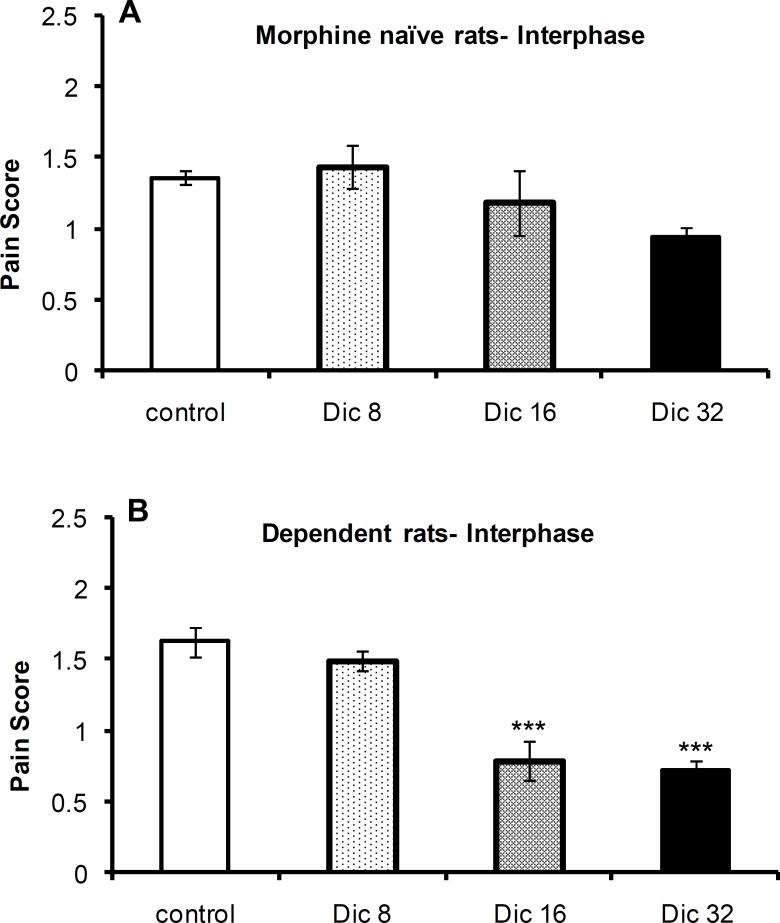
Pain scores during interphase of the formalin test in morphine-naïve (A) and morphine-dependent (B) groups. The data are expressed as the mean±SEM

The one-way ANOVA revealed no significant difference in pain scores [F(3,21)=1.56; p = 0.23; n = 6 or 4] among the control group and diclofenac-treated groups in morphine-naïve (Figure 2B) rats during the interphase of the formalin test; however, a significant difference was observed [F(3,23)=26.1; p < 0.0001; n = 6] among these groups in morphine-treated animals (Figure 2A). A Tukey’s post-test revealed a significant difference between both 16 mg/kg (p < 0.0001) and 32 mg/kg (p < 0.0001) dosage groups compared with the control group. 

*The antinociceptive effects of diclofenac in morphine-dependent or -naïve rats during phase II of the formalin test*

**Figure 3:**
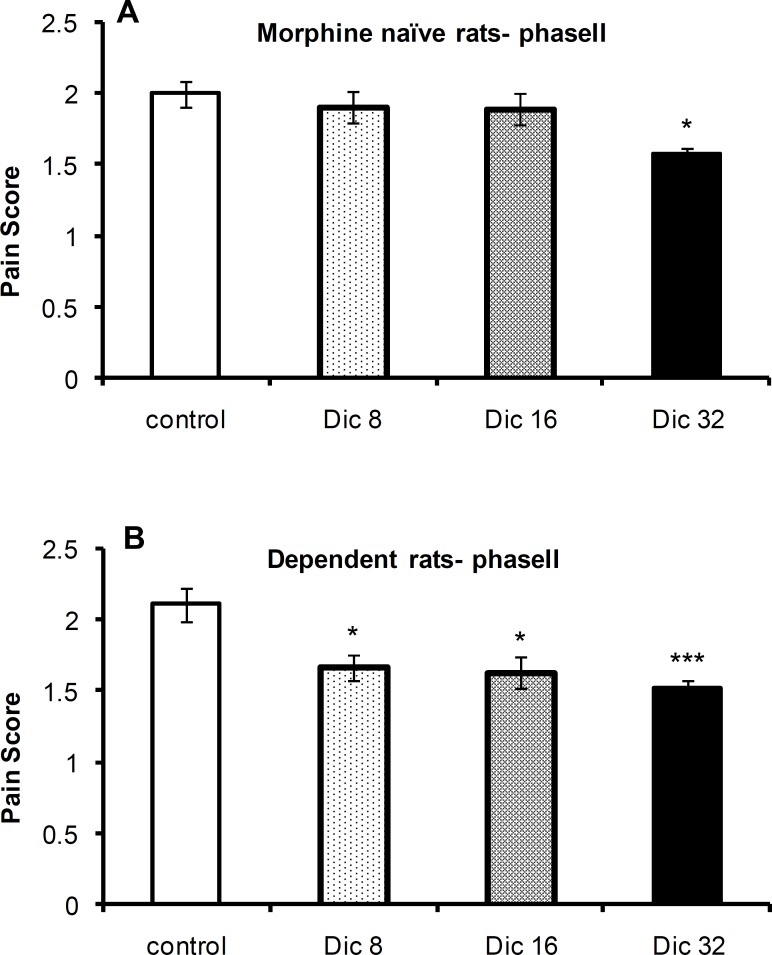
Pain scores during phase II of the formalin test in morphine-naïve (A) and morphine-dependent (B) groups. The data are expressed as the mean±SEM

The one-way ANOVA revealed a significant difference in pain scores between the control group and diclofenac-treated groups in morphine-naïve [F(3,21)=4.5;P=0.016; n=6 or 4] and morphine-treated [F(3,23) =7.1; p = 0.002; n = 6] rats during phase II of the formalin test. A Tukey’s post-test revealed a significant difference between the 8 mg/kg (p < 0.05), 16 mg/kg (p < 0.05), and 32 mg/kg (p < 0.01) groups and control group of morphine-treated animals. In morphine-naïve rats, a significant difference was only observed between the 32 mg/kg (p < 0.05; n = 6) group and control group (Figures 3A and 3B, respectively). 

## Discussion

In this study, we have assessed the antinociceptive effects of the NSAID diclofenac sodium on behavioral responses of morphine-treated rats subjected to formalin test. In this model. the source of pain is tissue damage, which makes it the most similar to clinical pain (26, 35). Formalin injection into the hind paw of rodents evokes two phases of pain: Phase I, which consists of neurogenic nociception, is a result of the direct stimulation of nociceptors by formalin. The pain message is transmitted through C fibers to the dorsal horn of the spinal cord after substance P is secreted and acts as a neurotransmitter; and Phase II, which consists of inflammatory-induced pain wich is a result of i) release of serotonin, histamine, bradykinin and prostaglandins from the tissue that is damaged by formalin, and ii) the plastic changes in the spinal synapses that are involved in nociception transmission (26, 36-40). During a 10 min period between phases I and II, which is called the interphase, a significant drop in nociceptive behaviors has been observed. Although some studies have suggested that this is due to the activation of the endogenous analgesic system (27, 41-43), the exact defined mechanism of the interphase remains unclear. 

The result of this study revealed that diclofenac does not affect neurogenic nociception during phase I of the formalin test in either morphine-naïve or -dependent animals. Although diclofenac and other NSAIDs have been extensively shown to be ineffective in attenuating the nociceptive behaviors of the early acute phase of the formalin test (44-46), this has not been observed in morphine-dependent rats. It appears that diclofenac has no significant effect on acute pain during phase I of the formalin test because it does not affect transduction in nociceptors and/or transformation in afferent pain fibers. 

The results of this study suggest that during the interphase of the formalin test, Diclofenac leads to potentiation of pain suppression in both morphine-dependent and -naïve rats. Although several previous studies have reported the antinociceptive effects of diclofenac and other NSAIDs on the nociceptive phases of the formalin test (44, 46, 47), this study is the first to suggest a role for diclofenac during the interphase. Since the interphase has not previously been well studied (41), the exact mechanism of nociception suppression during this phase has not been well understood. Electrophysiological recordings from C and A*δ *afferent fibers that had been stimulated with formalin also showed a three-stage activity pattern in which a single stage of attenuation of firing was identified between two stages of bursting activity (48, 49). This observation implied that a possible mechanism of interphase pain suppression is the inherent feature of pain afferent fibers that takes place passively. However, other studies suggest that an endogenously active inhibitory process may be responsible for pain suppression during the interphase (27, 41, 42, 43, 51-53). Although the anatomical substrate of this active suppression mechanism has not yet been clearly identified, current evidence suggests that both non-opioid (42, 51) and opioid (28) neurotransmitter systems are involved. In this study, it was difficult to explain the potentiation of spontaneous antinociception by diclofenac during the interphase of the formalin test in morphine-dependent rats. Because diclofenac is a non-selective COX enzyme inhibitor (54, 55), products of the COX pathways are likely involved in the non-opioid-dependent mechanism of pain suppression during the interphase. Gaumond *et al. *have provided evidence that underscores the involvement of the non-opioid-dependent mechanism of pain suppression in male rats by observing that the administration of naloxone in male rats did not influence the interphase during the formalin test, while in contrast, naloxone completely blocked the interphase in female rats (28).

In this study, diclofenac was also found to alleviate inflammatory pain during phase II of the formalin test both in morphine-dependent and -naïve animals. Many previous studies have reported that diclofenac and other NSAIDs have an antinociceptive effect on consistent inflammatory pain (45, 46). However, in this study, the antinociceptive effect of diclofenac on morphine-dependent rats started at lower doses than for morphine-naïve rats. Although the increased sensitivity of morphine-treated rats to the antinociceptive effect of diclofenac may be significant, the exact mechanism could not be determined. 

Most previous studies have associated morphine-induced biological effects with the increased activity of eicosanoid systems in both peripheral tissues and the CNS. Arachidonic acid metabolites such as prostaglandins interfere with the induction of morphine dependence in guinea pig ileum, which has been used as an *in-vitro *model of morphine dependence (56, 57). Both substance P and calcitonin gene-related peptide (CGRP) are involved in development of physical dependence to opioids, and their effects are mediated by prostaglandins (58-61). It has also been suggested that the products of eicosanoid pathways are involved in the development of tolerance to antinociceptive effects of morphine (62). Likewise, the intrathecal administration of a COX inhibitor has been shown to attenuate the tolerance to the antinociceptive effects of morphine by modulating of NMDA receptors and the NO system (40, 60). Furthermore, some evidence has suggested that an increase in sensitivity to pain that occurs during prolonged opioid exposure could be mediated through the products of COX pathways. Tumbaga *et al*. have reported that four hours of morphine sulfate infusion led to an increase in both the expression of COX_1_-mRNA in brain and prostaglandin E_2_ in sagittal sinus vein (63). In addition, morphine has been found to have an excitatory effect on the synthesis and secretion of PGs in peripheral tissue (64). While a number of mechanisms have been proposed to explain how the antinociceptive effects of diclofenac are induced at peripheral and central levels, the main mechanism by which diclofenac and other NSAIDs exert an antinociceptive effect is the inhibition of COX production pathway mediators in injured tissues (65-67). Therefore, the potent antinociceptive effect of diclofenac that was observed in our study on persistent pain in morphine-treated rats could result from the suppression of the production of normally overexpressed PGs in peripheral tissue and central nervous system. Since diclofenac is a non-selective COX1 and COX2 inhibitor, our findings concerning COX1 and COX2 interaction with morphine have not been helpful. Therefore, in order to produce more informative information over interaction mechanism, it is recommended to conduct complementary investigations using selective COX1 (such as mofezolac) or COX2 (such as celecoxib) inhibitors.

A clinically common pain treatment strategy is the administration of a combination of opioids along with NSAIDs (68-70). In addition to the synergistic effects that have been reported with this type of combinatory treatment, adding an NSAID to the pain control regimen may result in a significant reduction in the amount of opioids required (70) and could consequently reduce the opioid-related side effects (e.g., respiratory depression, increased pain sensitivity, and constipation).

## Conclusion

In this study, the antinociceptive effects of diclofenac were tested on rats that had free access to morphine-containing water up to 30 min prior to the experiment. Our findings clearly showed that morphine-dependent rats that consume a maintenance dose of morphine displayed a stronger reaction to the antinociceptive effects of diclofenac.

In conclusion, these results show that diclofenac results in potentiation of the pain suppression during the interphase and attenuation of nociceptive behaviors during phase II of the formalin test in morphine-treated rats. Although the endogenous analgesic system is suppressed in these animal, and they become tolerant to the antinociceptive effects of morphine, their sensitivity to the antinociceptive effect of diclofenac is increased as an adaptive response. Further research is required to elucidate the mechanisms of this response.
